# Chorea After MR-Guided Focused Ultrasound Thalamotomy

**DOI:** 10.5334/tohm.1085

**Published:** 2025-12-02

**Authors:** Sara J. Hooshmand, Tina Liu, Rushna Ali, Timothy Kaufmann, Vance Lehman, Bryan Klassen, Lauren Jackson

**Affiliations:** 1 Department of Neurology, Mayo Clinic, Rochester, Minnesota, USA; 2 Department of Neurological Surgery, Mayo Clinic, Rochester, Minnesota, USA; 3 Department of Radiology, Mayo Clinic, Rochester, Minnesota, USA

**Keywords:** MR-guided FUS, Chorea, essential tremor (ET), STN

## Abstract

**Background:**

Magnetic resonance-guided focused ultrasound thalamotomy (MR-FUS) is a promising, noninvasive treatment for medically refractory essential tremor (ET). It is well tolerated, with the most common side effects being sensory and gait disturbances.

**Case Report:**

A 69-year-old man presented with orofacial dyskinesias, left hemichorea, and motor impersistence 1 week after MR-FUS of the right ventralis intermedius nucleus for ET. MRI brain demonstrated right ventral thalamus T2 hyperintensity with inferolateral extension abutting the subthalamic nucleus (STN).

**Discussion:**

Chorea is a rare side effect of MR-FUS, but may be present with inferolateral lesions extension to the STN, disrupting the indirect pathway.

Essential tremor (ET) is one of the most common movement disorders, with an estimated prevalence of 0.9% [1]. Although ET is not life-threatening, it can lead to significant functional and social impairment [2]. First-line treatments typically include beta-blockers and anticonvulsants; however, these medications often provide limited benefit and may be poorly tolerated due to adverse effects or contraindications [2,3].

Deep Brain Stimulation (DBS) has been a well established treatment option for patients with ET for many decades [4]. However, patients may hesitate to proceed with DBS due multiple factors, including invasiveness, maintenance, and preference for noninvasive alternatives [5]. In 2016, magnetic resonance-guided focused ultrasound thalamotomy (MR-FUS) emerged as a promising, noninvasive treatment option for patients with medically refractory ET [6] Complication rates associated with MR-FUS are relatively low, with the most common side effects including sensory disturbances and gait imbalance, typically related to the location of the thalamotomy lesion [6,7]. Perilesional edema commonly occurs in the setting of MR-FUS, typically transient, arising within the first week and resolving within a month [8,9]. It is often associated with larger lesion volume, higher power delivery, and a rapid rise in tissue temperature [10]. In this report, we present the case of a 69-year-old male who developed hemibody chorea following MR-guided focused ultrasound.

## Case Report

A 69-year-old left-handed man presented to the movement disorders clinic for evaluation of chronic (10 year) left upper extremity tremor. The tremor significantly interfered with activities of daily living and was unresponsive to multiple medication trials. His past medical history was notable for depression, managed with aripiprazole 5 mg daily and sertraline 50 mg daily for the past 5 years. Family history was notable for first and second degree relatives with ET.

On pre-procedure examination, the patient’s tremor severity was assessed using the Fahn-Tolosa-Marin Tremor Rating Scale (FTM-TRM). He scored 6 for moderate to severe left predominant postural and kinetic tremor (1 right postural tremor, 3 left postural tremor with two-second latency period, and 2 for moderate left kinetic tremor). There was slight reemergence of tremor during ambulation, but no evidence of rest tremor, rigidity, or bradykinesia with finger tapping or toe tapping bilaterally (Video 1). For pre-procedural planning, he underwent electrophysiological testing, which confirmed a consistent left sided 5–6 hertz postural kinetic tremor without any rest component, consistent with underlying ET. To ensure there was no significant medication component, Aripiprazole was discontinued and repeat evaluation six months later did not result in any notable improvement in his tremor. He remained off Aripiprazole following this.

**Video 1:** Patient movements pre and post MR-FUS. Prior to MR-guided focused ultrasound thalamotomy, examination showed left upper extremity postural and kinetic tremor. Post procedure exam revealed orofacial dyskinesias and left hemibody choreiform and dystonic movements. Reevaluation after approximately eight weeks of tetrabenazine therapy demonstrated marked improvement in the choreiform and dystonic movements.

**Figure 1:**
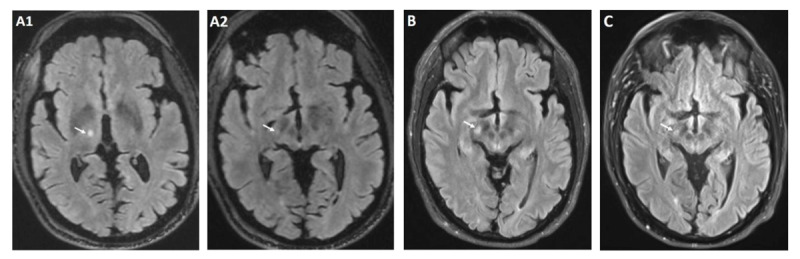
Brain MRI T2 FLAIR post procedure. Immediately post procedure lesion at Vim **(A1)** with very slight extension to STN **(A2)** 1 month after procedure and onset of chorea with increased signal intensity involving the right STN **(B)** 8 months after procedure with near resolution of STN signal hyperintensity **(C)**.

Given the medically refractory nature of his essential tremor, he underwent MR-FUS targeting the right ventral intermediate nucleus (Vim) of the thalamus (Figure 1A). The skull density ratio (SDR) was 0.63. The lesion site was localized 14 mm lateral to the midline, 25% of the AC-PC distance anterior to the posterior commissure (approximately 7.2 mm anterior) and 2.0 mm superior to the AC-PC plane. A total of six sonications were delivered, with a maximum energy of 9,444 Joules. Intraoperative temperatures ranged from 44°C to 58°C. The patient was assessed throughout the procedure for tremor improvement and potential adverse neurological effects, including paresthesia, weakness, ataxia, and dysarthria. Immediate post-sonication MRI demonstrated a well-circumscribed lesion within the right Vim, measuring approximately 240 mm^3^ in volume and located 3.63 mm from the margin of the lesion to the subthalamic nucleus (STN).

The procedure was uncomplicated, and he continued to remain off Aripiprazole in the post procedure period. Within one-week post-procedure, the patient reported involuntary movements of the left upper extremity. Due to concerns for post-procedural edema, an 8-day dexamethasone taper was initiated; however, there was no notable benefit. At the one-month follow-up, brain magnetic resonance imaging (MRI) demonstrated T2 hyperintensity in the right ventral thalamus, with signal abnormality extending inferolateral to abut the STN (Figure 1B). Neurological examination demonstrated marked improvement of the left-predominant tremor, with the FTM-TRS score decreasing from 6 pre-procedure to 1, indicating only mild kinetic tremor. However, there was new orofacial dyskinesias and left hemichorea involving proximal and distal muscle groups. There was also dystonic posturing of the left hand, which was present at rest and during ambulation (Video 1). These movements significantly impaired the patient’s quality of life, causing hand tightness from dystonic posturing and lip soreness due to oral dyskinesia.

Treatment options were carefully considered given the notable impact of these movements on the patient’s quality of life. After a thorough discussion of the potential benefits and risks, particularly in the context of the patient’s history of depression, tetrabenazine was initiated for symptomatic improvement. The decision was supported by the patient’s well-controlled mood symptoms, the medication’s established efficacy, and the availability of close clinical monitoring. Tetrabenazine 25 mg twice daily was initiated, resulting in significant improvement of the left hemibody movements, with only residual choreiform and dystonic movements of the left upper extremity and orofacial muscle groups (Video 1). This improvement was sustained through the eight-month follow-up. At that time, follow-up MRI showed a decrease in STN hyperintensity (Figure 1C). Despite this radiographic improvement, tapering tetrabenazine led to recurrence of left hemibody chorea, necessitating initiation of the previous dose.

## Discussion

Our patient developed left hemibody chorea following MR-FUS targeting the Vim for essential tremor secondary to lesional extension into the STN. Disruption of the STN is a well-established cause of hyperkinetic movement disorders such as chorea and ballism, typically seen in the context of vascular insults [11,12]. However, it is rarely recognized as a potential complication of MR-FUS, with only a few cases reported to date [13,14].

The manifestations of chorea and dystonia in our case, as well as other cases with STN lesions, are attributed to disruption of the indirect basal ganglia pathway. When the STN is compromised, there is reduced inhibitory output of the indirect pathway, leading to excessive thalamic stimulation of the cortex, ultimately producing involuntary movements [11].

Interestingly, our patient’s chorea persisted despite radiological improvement of the lesion, requiring continued treatment with tetrabenazine. We hypothesize that this may be partially related to the patient’s pre-procedural use of aripiprazole which posed a risk for delayed tardive dyskinetic movements that may have been unmasked by lesional disruption of the indirect pathway, even if only transiently. Aripiprazole carries a cumulative, dose-dependent effect on basal ganglia circuits may induce maladaptive plasticity and altered dopamine receptor sensitivity, leading to delayed and persistent symptoms even after discontinuation [15]. In retrospect, the atypical tremor features, such as delayed re-emergence and a rotatory component, may reflect the effects of chronic dopamine-blocking exposure rather than pure ET phenotype. We suspect that the patient’s prolonged use of anti-dopaminergic therapy increased his susceptibility to chorea and dystonia through inhibition of the indirect pathway, which was likely unmasked by lesional disruption and persisted despite radiographic improvement [15,16].

While MR-FUS is an effective and generally well-tolerated treatment for essential tremor, our case highlights a rare but clinically significant complication [7]. Although uncommon, choreiform and dystonic movements should be recognized as a potential adverse effect when the inferolateral Vim is targeted, due to its proximity to the STN. The persistence of these symptoms in our case is unique and highlights a potential predisposition to hyperkinetic movement disorders in those who are on chronic antidopaminergic medications. This underscores the importances of thoughtful patient selection and counseling regarding potential adverse effects as the clinical use of MR-FUS continues to grow. Awareness of this rare but potentially treatable complication is essential. Careful planning, precise targeting, and close post-procedural monitoring may help mitigate the risk and ensure optimal outcomes for patients undergoing this therapy. Future studies could investigate factors that predispose patients to these rare adverse effects, as well as strategies for initiation and management of dopamine-depleting agents, like tetrabenazine. With appropriate patient selection, this approach may be beneficial; however, thorough patient education, close follow-up, and multidisciplinary approach should be implemented given risk for worsening depression and suicidality.

## Ethics and Consent

The patient appearing in the video provided informed consent. Authorization for videotaping and publication of the video was obtained.
